# Prevalence, features and health impacts of eating disorders amongst First-Australian Yiramarang (adolescents) and in comparison with other Australian adolescents

**DOI:** 10.1186/s40337-020-0286-7

**Published:** 2020-03-12

**Authors:** Adam Burt, Deborah Mitchison, Elizabeth Dale, Kay Bussey, Nora Trompeter, Alexandra Lonergan, Phillipa Hay

**Affiliations:** 1 School of Medicine, Western Sydney University, Sydney, Campbelltown Australia; 2 Translational Health Research Institute (THRI), School of Medicine, Western Sydney University, Sydney, Campbelltown Australia; 3 Centre for Emotional Health, Department of Psychology, Macquarie University, North Ryde, New South Wales Australia; 4 Ngarruwan Ngadju First Peoples Health and Wellbeing Research Centre, Australian Health Services Research Institute and Illawarra Health and Medical Research Institute, School of Psychology, University of Wollongong, Wollongong, New South Wales Australia; 5 Centre for Emotional Health, Department of Psychology, Macquarie University, Sydney, Australia; 6 Campbelltown Hospital, SWSLHD, Sydney, Campbelltown Australia

**Keywords:** Feeding and eating disorders, Adolescents, Prevalence, Oceanic ancestry, Group, Aboriginal and Torres Strait islander

## Abstract

**Background:**

This study aimed to support previous research conducted with First-Australians (FA) by establishing the prevalence of eating disorders, and their demographic distribution and burden in adolescent First-Australians compared to other-Australians (OA).

**Methods:**

Data were used from the baseline survey of the EveryBODY Study, a longitudinal investigation of eating disorders among Australian adolescents. Of the 5068 participants included, 402 (8%) identified as FA, 4586 (90.5%) identified as OA. Diagnosis of eating disorders was based on the Diagnostic and Statistical Manual version 5. Socioeconomic status and measures of impairment were assessed using validated instruments. Body mass index was calculated based on self-reported weight and height. Statistical analyses used data weighted to the distribution of gender in adolescents in New South Wales in the 2016 Australian Census. Chi-square tests were performed to determine prevalence of eating disorders amongst FA and to compare to OA. ANOVA and logistic regression analyses where conducted to examine the moderation effect of sociodemographic status, measures of impairment and FA status on the distribution of eating disorders**.**

**Results:**

The prevalence rates for eating disorder diagnoses where similar for FA and OA with the exception of Night eating Syndrome (OSFED-NES), which occurred in 7.14% (95%CI 4.81–10.49) of FA vs. 3.72% (95%CI 3.17–4.36) in OA. The greater prevalence of OSFED-NES in FA was largely explained by poorer psychosocial quality of life amongst FA.

**Conclusion:**

Eating disorders are common amongst First-Australian adolescents and are associated with poor psychosocial quality of life. These findings are consistent with previous research conducted with First-Australian adults. There is a need to screen for eating disorders amongst First-Australian adolescent girls and boys.

## Plain English summary

This study aimed to support previous research conducted with First Australian adults by establishing the prevalence, distribution and burden of eating disorders amongst First Australian adolescents, and compare this to other Australian adolescents. Data were used from the EveryBODY study, a survey of eating disorders amongst Australian adolescents. Of 5068 adolescents who completed the survey, 8% (402) were First Australians. All participants answered questions on basic demographic information, eating disorders, and impairment. The prevalence of eating disorders amongst First Australian adolescents was 28.62%, which was significantly greater than among other-Australian adolescents (21.68%). The greater prevalence of eating disorders amongst First Australians was largely driven by the higher prevalence of Night Eating Syndrome (7.14% amongst Fist Australians vs. 3.72% amongst other Austalians) and poorer psychosocial quality of life.

## Background

First-Australians have poorer physical and mental health than other-Australians in all age groups, including adolescents [1, 2]. First-Australian adolescents have a two-fold greater all-cause mortality rate than other-Australian adolescents, mostly due to suicide and road traffic injuries. Eighty percent of this mortality has been reported as potentially avoidable within the current Australian healthcare system [2]. First-Australian adolescents also have a greater burden of non-fatal disease [1]. Approximately 20% of First-Australian adolescents are obese and 40% are either obese or overweight, amongst the highest rates for adolescents in the world [2]. First-Australian adolescents aged 18–24 years suffer twice the rate of high to very high psychological distress of other-Australian adolescents [2], not surprisingly First-Australian adolescents also have a significantly greater burden of mental disorders including: conduct disorder, intentional self-harm, anxiety disorders, depressive disorders and alcohol use disorders [1]. Hospital separations for psychosis, alcohol and other substance use disorders are three-fold higher in First-Australian adolescents compared to other-Australian adolescents [2]. These statistics are alarming, as First-Australians are on average younger than other-Australians (21.8 years vs. 37.6 years). First-Australian adolescents also make up a greater proportion of their respective population compared to other-Australian adolescents (37.7% vs. 20.4%) [1, 2].

Adolescence is an important developmental period characterised by a range of physical, cognitive and psychosocial changes, [3] it is also the time when eating disorders are most likely to emerge [4]. Evidence from an Australian prospective cohort study suggests that the prevalence of eating disorders amongst Australian adolescents is 6.2% for one of the three major eating disorders (i.e., anorexia nervosa, bulimia nervosa, binge eating disorder) and as high as 22.2% when including atypical and subthreshold eating disorders [5]. Yet the prevalence of eating disorders amongst First-Australian adolescents remains unknown.

There is a lack of good quality research into mental disorders amongst First-Australians generally, [6, 7] but especially so in regards to eating disorders. A systematic review into the prevalence of mental disorders amongst First-Australians did not identify any studies that assessed eating disorders [7]. A systematic synthesis of First-Australian Adolescent health population data resorted to the use of hospital separation data in order to measure the prevalence of eating disorders, as well as other mental disorders, as there were insufficient data available from surveys. The latter study did not describe the prevalence of individual eating disorders [2]. The research that is available on eating disorders amongst First-Australians is conflicting. An example of this was seen in the previously mentioned systematic synthesis of First-Australian adolescent health population data, which suggested that eating disorders are more common amongst other-Australian compared to First-Australian adolescents and are more common amongst urban compared to rural First-Australian adolescents [2]. These data conflict with research suggesting eating disorders occur at similar rates across urbanicity and socioeconomic status [8, 9]. Further to this, emerging research indicates that eating disorders are actually more common amongst First-Australian compared to other-Australian adults and are associated with high levels of overvaluation of weight and shape concerns. The same study found the higher prevalence of eating disorders amongst First-Australians’ was largely associated with the younger age, higher BMI and poorer mental health of First-Australians [10].

In support of this, previous Australian population health and body image research has also suggested that these explanatory variables (younger age, higher BMI and mental health impairment), as well as physical health impairment and body image disturbance are also greater amongst First-Australian compared to other-Australian adolescents. This line of research thus indicates the potential of elevated risk of eating disorders among First-Australian adolescents. For example, an Australian study into media influences on body image and disordered eating among First-Australian adolescents found that First-Australian adolescents were more dissatisfied with their weight and engaged in more activity to change their weight and shape compared to other-Australian adolescents. First-Australian adolescents in this study were also more sensitive to media messaging to lose weight, despite receiving fewer media messages [11]. It should be noted however that this study was conducted in the early 2000s and newer research is needed to determine the impacts of today’s widespread personalised and multimedia messaging on First-Australian adolescents’ perceptions.

Based on the recent research findings into eating disorders amongst First-Australian adults, we suspect that the relatively larger pool of young people in the First-Australian community, burdened with other risk factors such as body image disturbance, poorer mental and physical health and higher rates of overweight and obesity may place them at greater risk of eating disorders.

## Gap and aim

It is important to investigate the prevalence of eating disorders amongst First-Australian adolescents, especially given the sparse evidence available and the evidence to date suggesting a potentially elevated risk of eating disorders among this group. Further, this line of research forms a priority area for the Australian Government. The National Aboriginal and Torres Strait Islander Health plan 2013–2023 recognises evidence-based practice as important to guide the health care of First-Australians and asserts a need for more high quality research conducted in partnership with First-Australian communities to achieve this. The plan highlights adolescent, youth and mental health as specific priority areas for research and improvements [12, 13]. Thus this study aimed to support previous research conducted with First-Australians by establishing the prevalence of eating disorders, and their demographic distribution and burden in adolescent First-Australians compared to other-Australians.

## Methods

### Procedures and participants

Data were used from the baseline survey of the EveryBODY Study. The EveryBODY study is a longitudinal investigation of eating disorders among Australian adolescents. Sampling procedures have been detailed elsewhere [5, 14]. In brief, four independent and nine government schools, from a broad range of socioeconomic advantage, participated. All parents and students received information about the study over a period of four weeks using multiple methods of dissemination, and a passive parental consent procedure was used, whereby parents could opt out their child from the study. Students who provided consent were given the online survey to complete at school. Participants were offered the chance to enter a prize draw to win one of ten gift vouchers, and the schools received a general wellbeing report based on their students’ data.

Of the 5280 students who commenced the survey, *n* = 123 were excluded due to: completion of < 10% of the survey (*n* = 39), non-serious responses to open-ended questions (*n* = 79), incorrect coding (*n* = 4) and withdrawn consent (*n* = 1). Of the remaining participants, *n* = 402 (8%) identified as First Australian (*n* = 355 of Aboriginal origin, *n* = 28 of Torres Strait Islander origin and 19 of both Aboriginal and Torres Strait Islander origin), *n* = 4586 (90.5%) identified as other-Australian, and *n* = 80 (1.6%) declined to provide a response to the question regarding Indigenous status.

## Diagnostic status and measures

### Sociodemographic questions

A socio-economic index for area (SEIFA) score was calculated based on the residential postcode of participants. A SEIFA score is an Australian Bureau of Statistics (ABS) product used to rank areas in Australia based on relative socioeconomic advantage or disadvantage [15]. Other demographic information collected included age, school grade, gender, sex, and country of birth.

### Eating disorder diagnoses

The operationalisation of the eating disorder diagnoses that were assessed in this study has been published previously [5], and an adapted Table of this operationalisation is included in the Supplementary material. Diagnoses assessed included the three major eating disorders (anorexia nervosa, bulimia nervosa, binge eating disorder), the five Other Specified Feeding and Eating Disorders (OSFED; atypical anorexia nervosa, subthreshold bulimia nervosa, subthreshold binge eating disorder, purging disorder, night eating syndrome) and Unspecified Feeding and Eating Disorder (UFED). Most symptoms were captured by items of the Eating Disorder Examination Questionnaire (EDE-Q), which assesses the presence and severity of cognitive and behavioural eating disorder symptoms and features [16]. This questionnaire has previously been validated in Australian adolescent boys and girls and demonstrates sound reliability [17]. Items used in this study included the behavioural frequency items (self-induced vomiting, laxative misuse, driven exercise, and binge eating), and the Likert-type items that comprise the combined weight and shape concern subscales. As the frequency of behaviours were only assessed over the past 1 month (not the 3 months duration required for bulimia nervosa and binge eating disorder), we use the term “probable” for these diagnoses. Cronbach’s alpha for the combined weight and shape concern subscale in the present study was 0.96 for both First-Australian and other-Australian adolescents.

Participant’s self-reported current weight and height, which was converted to age and gender adjusted body mass index (BMI) percentiles for children and adolescents. A BMI percentile < 10 was used for the underweight criterion of anorexia nervosa, as this cut-off has most frequently been used in adolescent epidemiological studies of DSM-5 anorexia nervosa [18–21]. Three items from the Night Eating Questionnaire (NEQ) [22] were used to assess symptoms of night eating syndrome, including proportion of daily food intake consumed following supper, nocturnal eating (eating after going to bed), and awareness during nocturnal eating. The NEQ has been validated in adolescents and is superior to parent report [23].

Several additional questions were developed by the researchers to capture frequency of additional extreme weight control behaviours (fasting, strict dieting, detoxes, insulin misuse, other drug use for weight loss), distress associated with binge eating, and additional diagnostic binge eating disorder features (e.g., eating faster than usual, eating alone due to embarrassment). Participants were also asked about any recent weight loss in the past 4 weeks to assess atypical anorexia nervosa.

### Quality of life impairment

Scores from the Paediatric Quality of Life Scale (PedsQL) SF15 [24, 25] were used to measure quality of life impairment. The 12 items from the physical functioning, emotional functioning, and social functioning subscales were included in the survey. Items ask participants to indicate on a Likert type scale how true a series of statements are of them in the past 4 weeks. Scores are reversed and transformed on a 0–100 scale, such that higher scores indicate higher functioning. Subscale scores are derived as the mean of the items for that scale. For the purposes of this study we combined the emotional and social functioning scales to create a psychosocial subscale. The PedsQL SF15 has evidence of good reliability and validity in previous studies of adolescents [25]. Cronbach’s alphas in the current study sample for the physical functioning subscale was 0.89 and 0.84 for First Australian and other-Australian adolescents respectively, and for the psychosocial functioning subscale was 0.91 and 0.90 for First-Australian and other-Australian adolescents respectively.

### Statistical analysis

Analyses used data weighted to the distribution of gender in adolescents in New South Wales in the 2016 Australian Census. A series of Chi-square tests were performed to determine the prevalence of eating disorders among First-Australian adolescents and to compare this to other-Australian adolescents. This was followed by 2 × 2 ANOVAs and logistic regression analyses to examine the moderation effect of First-Australian status on the sociodemographic (dependent variables: age, sex, SEIFA score, BMI percentile) distribution of eating disorders. Additional 2 × 2 ANOVAs and logistic regressions were employed to test the moderation effect of First-Australian status on the association between eating disorders and specific symptoms (dependent variables: weight and shape concerns, binge eating, self-induced vomiting, laxative use, dietary fasting, driven exercise) and impairment (psychosocial quality of life and physical quality of life impairment). Finally, four logistic regressions were performed with current eating disorder status as the outcome. In regression one, First-Australian status was the only predictor in the model; in regression two, sociodemographic variables (age, gender, SEIFA score, and BMI percentile) were added to the model; in regression three, eating disorder symptom variables (weight and shape concerns, binge eating, self-induced vomiting, laxative use, fasting, driven exercise) were added to the model; in regression four, impairment variables (psychosocial and physical quality of life impairment) were added to the model. The intention of these analyses was to examine the extent to which First-Australian status remained independently associated with the likelihood to meet criteria for a current eating disorder in the presence of other known correlates. In these analyses, SEIFA score was divided by 100 (the normed SD), BMI percentile was divided by 10, and weight/shape concerns and psychosocial and physical quality of life impairment scores were standardised, to allow more meaningful interpretation of the adjusted odds ratios.

## Results

### Prevalence of eating disorders amongst first-Australian adolescents

Overall, the prevalence of any eating disorder (i.e., major eating disorder, OSFED or UFED) was higher among First-Australian adolescents (28.6%) compared to other Australian adolescents (21.7%). Of note, eating disorder prevalence was also very high among adolescents who chose not to specify their First-Australian status (32.8%), however possibly due to reduced power in this smaller sub-group, this proportion did not differ statistically from either the First Australian or other-Australian groups. Further analyses excluded participants who did not disclose their First-Australian status. Table 1 displays the point prevalence for each eating disorder, separated by First-Australian status. As can be seen, there was a higher prevalence of any eating disorder and OSFED in First-Australian adolescents compared to other adolescents. This seemed to be largely explained by a higher prevalence of night eating syndrome (7.1% vs 3.7%, respectively) and purging disorder (5.1% vs 3.1%, respectively), although the latter neared but did not reach statistical significance. Other specific eating disorder diagnoses also trended to be more prevalent among First-Australian adolescents however these differences did not reach or come close to statistical significance.

**Table 1:** One-month prevalence of eating disorders in First-Australian (FA) and other-Australian (OA) adolescents

	First-Australian Adolescents	Other-Australian Adolescents	
	% (95% CI)	% (95% CI)	χ^2^ (1), *p*
Any Eating Disorder (Major, OSFED, UFED)	28.62 (23.83–33.94)	21.68 (20.38–23.03)	**7.84, .005**
Major Eating Disorder	7.53 (5.15–10.88)	6.07 (5.38–6.85)	.13, .288
Anorexia Nervosa (AN)	.89 (.30–2.59)	.66 (.45–.96)	0.49, .401†
Probable Bulimia Nervosa (BN)	5.11 (3.21–8.02)	4.45 (3.86–5.13)	.31, .579
Probable Binge Eating Disorder (BED)	1.80 (.83–3.87)	.96 (.70–1.31)	2.16, .142
Other Specified Feeding and Eating Disorders (OSFED)	15.22 (11.71–19.55)	10.77 (9.83–11.78)	**5.97, .015**
Atypical Anorexia Nervosa (OSFED-AAN)	3.92 (2.30–6.58)	2.69 (2.23–3.23)	1.71, .191
Subthreshold Bulimia Nervosa (OSFED-SBN)	1.50 (.64–3.47)	2.21 (1.8–2.71)	.74, .390
Subthreshold Binge Eating Disorder (OSFED-SBED)	.30 (.05–1.68)	.32 (.19–.55)	<.01, .952
Purging Disorder (OSFED-PD)	5.11 (3.21–8.02)	3.12 (2.63–3.7)	3.82, .051
Night Eating Syndrome (OSFED-NES)	7.14 (4.81–10.49)	3.72 (3.17–4.36)	**9.12, .003**
Unspecified Feeding or Eating Disorder (UFED)	5.32 (3.30–8.46)	4.47 (3.85–5.19)	.46, .500

Note. Significant effects are highlighted in bold text. Based on weighted data. † based on Fishers Exact test due to low cell numbers. Total N for prevalence analyses varied for each diagnosis, dependent on the missingness of diagnostic data (AN: *N* = 4425; BN: *N* = 4401; BED/PD: *N* = 4399; AAN: *N* = 4388; sBN/sBED = 4398; NES: *N* = 4220; UFED: *N* = 3989; Major ED: *N* = 4401; OSFED: *N* = 4223; Any ED: *N* = 4036). The proportions of each diagnosis do not add up to the totals, except for the other-Australian category of Major eating disorders because where the DSM-5 allows it there is comorbidity (for instance between the OSFED diagnoses) – this is why the major eating disorder category proportion total is closer to the summation of the individual diagnoses within that category (the DSM-5 does not allow concurrent diagnosis between AN, BN and BED). The chi-square analyses are weighted, which slightly alters cell numbers

### Demographic distribution of eating disorders as moderated by first-Australian status

As can be seen in Table 2, although the sex distribution was similar in First-Australian and other-Australian adolescents in this sample, on average First-Australian adolescents were slightly younger, from lower socioeconomic backgrounds, and had a higher BMI percentile than other-Australian adolescents. Adolescents with eating disorders in general were more likely to be female, slightly older, and have a higher BMI percentile. Analyses of the interaction between First-Australian status and eating disorder status revealed that First-Australian status was not a significant moderator of the relationship between current eating disorder and age, sex, socioeconomic status, or BMI. These findings indicate that First-Australian and other-Australian adolescents with an eating disorder have similar sociodemographic distributions.

**Table 2:** Univariate associations between demographic and clinical correlates and First Australian (FA) and eating disorder (ED) status, and their interaction

	First Australian		Other Australian					
	ED	No ED	ED	No ED		Main Effect FA Status	Main Effect ED Status	Interaction Effect
	M (SD)				*df*	*F,η*_*p*_^*2*^, *p*		
Age	15.0 (1.3)	14.4 (1.5)	15.2 (1.4)	14.7 (1.4)	1, 4045	**7.35, .007, .002**	**25.40, .006, < .001**	.19, .66, < .001
Socioeconomic status	975.0 (42.4)	965.1 (42.1)	987.4 (40.9)	988.3 (42.3)	1, 3984	**40.11, < .001, .010**	2.53, .001, .112	3.64, .057, .001
BMI percentile	69.2 (27.6)	55.7 (31.7)	61.6 (29.7)	50.7 (30.7)	1, 4045	**9.95, .002, .002**	**36.69, .009, < .001**	.41, .521, < .001
Weight and Shape Concerns	4.0 (1.8)	0.9 (1.2)	4.0 (1.6)	1.0 (1.2)	1, 4045	.60, .440, < .001	**1235.15, .234, < .001**	.02, .893, < .001
Psychosocial quality of life impairment	55.7 (27.2)	79.8 (20.4)	58.5 (25.2)	82.3 (19.0)	1, 3972	3.65, .056, .001	**295.51, .069, < .001**	.02, .896, < .001
Physical quality of life impairment	72.6 (25.6)	85.4 (22.9)	77.6 (23.4)	89.0 (15.5)	1, 3972	**12.63, < .001, .003**	**101.16, .025, < .001**	.35, .555, < .001
	*n* (%)					OR (95% CI) FA adolescent	OR (95% CI) Current ED	β (s.e.), *p*
Female Sex	59 (67.8)	84 (38.5)	593 (73.3)	1329 (45.5)		1.32 (0.81, 2.81)	**3.30 (2.77, 3.93)**	0.01 (0.29), .965
Objective binge eating	27 (31.0)	11 (5.0)	273 (33.7)	147 (5.0)		1.00 (0.54, 1.88)	**9.54 (7.67, 11.87)**	−0.12 (0.40), .766
Fasting	24 (27.6)	13 (6.0)	249 (30.9)	129 (4.4)		1.38 (0.77, 2.49)	**9.68 (7.70, 12.17)**	−0.47 (0.39), .225
Self-induced vomiting	15 (17.4)	5 (2.3)	90 (11.1)	25 (0.9)		**2.88 (1.09, 7.61)**	**15.00 (9.51, 23.67)**	−0.49 (0.58), .394
Laxative use	6 (7.0)	4 (1.8)	45 (5.6)	22 (0.8)		2.62 (0.89, 7.70)	**7.80 (4.62, 13.17)**	−0.72 (0.71), .311
Driven exercise	8 (9.2)	7 (3.2)	123 (15.2)	95 (3.2)		1.01 (0.46, 2.21)	**5.40 (4.08, 7.13)**	−0.61 (0.55), .266

Note. Socioeconomic status measured by SEIFA score; weight and shape concerns measured with the Eating Disorder Examination Questionnaire combined Weight and Shape Concern subscales score; quality of life impairment measured with the Pediatric Quality of Life Scale; objective binge eating, fasting, self-induced vomiting and laxative use defined as > 4 episodes in the past 28 days; driven exercise defined as > 20 episodes in the past 28 days. *BMI* body mass index

### Clinical correlates of eating disorders and moderation by first-Australian status

As can be seen in Table 2, First-Australian adolescents in general reported greater physical quality of life impairment and more frequent self-induced vomiting but similar levels of other eating disorder symptoms and psychosocial quality of life. As expected, adolescents with eating disorders in general reported more eating disorder symptoms and greater impairment in quality of life than adolescents without an eating disorder. No moderating effects of First-Australian status were found, indicating that First-Australian and other-Australian adolescents with eating disorders report similar symptom profiles and impairment.

### Multivariate model of eating disorder correlates

As can be seen in Table 3, First-Australian status was a significant univariate correlate of current eating disorder status, with First-Australian adolescents around 1.5 times more likely to meet criteria for a current eating disorder compared to other-Australian adolescents. This effect remained significant even when the model accounted for sociodemographic variables and for behavioural and cognitive eating disorder symptoms. However, when measures of impairment were added to the model, First-Australian status was no longer independently associated with odds for current eating disorder, and this appeared to be largely explained by psychosocial quality of life impairment. Other factors that remained independently associated with meeting criteria for an eating disorder in the full multivariate model included all eating disorder symptoms and male sex.

**Table 3:** Multivariate associations between First Australian status, demographic and clinical correlates and eating disorder status

	Step 1	Step 2	Step 3	Step 4
	None	Socio-demographic	Socio-demographic + eating disorder symptoms	Socio-demographic + eating disorder symptoms + impairment
Variable	(Adjusted) Odds Ratio (95% CI)			
First Australian status	**1.44 (1.11, 1.87)**	**1.59 (1.20, 2.11)**	**1.55 (1.02, 2.35)**	1.49 (0.97, 2.29)
Age		**1.22 (1.16, 1.29)**	**1.08 (1.00, 1.17)**	1.01 (0.93, 1.10)
Female sex		**3.60 (3.03, 4.27)**	**0.67 (0.51, 0.86)**	**0.75 (0.57, 0.98)**
SES		1.02 (0.85, 1.23)	0.95 (0.73, 1.25)	1.05 (0.80, 1.38)
BMI-percentile		**1.14 (1.11, 1.17)**	0.98 (0.94, 1.02)	0.99 (0.95, 1.03)
Weight/shape concerns			**6.98 (6.04, 8.05)**	**6.18 (5.31, 7.20)**
Objective binge eating			**3.03 (2.25, 4.08)**	**2.95 (2.17, 3.99)**
Self-induced vomiting			**3.85 (2.01, 7.39)**	**2.48 (1.27, 4.86)**
Laxative use			**2.37 (1.03, 5.45)**	**2.89 (1.24, 6.78)**
Fasting			**2.03 (1.48, 2.79)**	**1.96 (1.41, 2.72)**
Driven exercise			1.37 (0.91, 2.07)	**1.55 (1.02, 2.37)**
Psychosocial quality of life impairment				**0.75 (0.65, 0.86)**
Physical quality of life impairment				0.93 (0.82, 1.06)

Note. Significant AORs are in bold text

Reference categories for categorical variables: other-Australian status, male sex, < 4 episodes of objective binge eating/self-induced vomiting/laxative use/fasting, < 20 episodes of driven exercise

Scale for continuous variables: years (age), standard deviation unity (SES, weight/shape concerns, psychosocial quality of life impairment, physical quality of life impairment), deciles (BMI percentile)

## Discussion

This study is the first to our knowledge to estimate the prevalence of eating disorders amongst First-Australian adolescents and supports previous research amongst First-Australian adults. Eating disorders are common amongst First-Australian adolescents. While overall, eating disorders were more common among First-Australian adolescents, the only specific diagnosis where First-Australian adolescents were statistically more likely to meet the diagnostic criteria was OSFED-NES, with other disorders being equally prevalent among First-Australian and other-Australian adolescents alike. The greater prevalence of eating disorders amongst First-Australian adolescents was largely explained by poorer psychosocial quality of life experienced more generally by First-Australian adolescents. This finding is in-line with eating disorder research suggesting poor quality of life is associated with eating disorders, and more specifically, recent research conducted with First-Australian adults indicating that mental health related quality of life impairment partly explained the higher prevalence of eating disorders among First-Australian adults. Eating disorders are well known to be associated with quality of life impairment [26]. There is evidence also that the link is bi-directional, in that while eating disorders impair quality of life, quality of life impairment in itself also increases the risk for developing eating disorders [27]. Thus, the poorer general quality of life among First-Australian adolescents may explain the greater prevalence of current eating disorders. First-Australians in this study also had lower socioeconomic status, which was also an explanatory variable for eating disorders.

As previously stated, there is a relatively larger pool of younger people in the First-Australian population compared to the other-Australian population, who are more socioeconomically disadvantaged and psychologically distressed, with poorer mental and physical health generally. In view of the similarity in findings of recent research conducted with First-Australian adults and adolescents, we suggest the higher prevalence of eating disorders amongst First-Australian adolescents and adults may be result of a relatively larger pool of people more vulnerable to mental disorders, including eating disorders. Given the differences between First-Australian groups culturally and the lack of research evidence, it is unclear whether specific cultural differences form part of the differences in eating disorder prevalence between First-Australians and other-Australians. It is unclear why OSFED-NES is specifically more common amongst First-Australian adolescents, whereas other eating disorders are not. One potential mechanism may be a greater prevalence of sleep problems among First-Australian children [28]. OSFED-NES is linked to insomnia and the belief that one needs to eat to initiate or maintain sleep [29]. Clearly further research is required to confirm and to understand these potential, and other, explanations for the differences found.

Eating disorders amongst First-Australian adolescents were associated with weight and shape concerns. However, unlike research amongst First-Australian adults, these concerns were not markedly greater than in the other-Australians group in univariate analysis. A possible explanation for this is that unlike previous research, this study focused on adolescents, which is the peak period for body image concerns, with all youth at heightened risk. Combining the current findings with previous findings in adults, it could be the case that the decline in weight and shape concerns typically found following the adolescent risky period either does not occur, or occurs to a lesser extent among First-Australian youth (i.e., they experience a prolonged period of risk compared to their other-Australians counterparts).

In the multivariate analyses (Table 3) the findings of the step 3 model were the reverse of what was found in the step 2 model, which included just sociodemographic variables, and where female sex was associated with greater odds for current eating disorder. However, when eating disorder symptoms and impairment were added to the model, it appears that female sex may be associated with eating disorder status due to the higher rates of eating disorder symptoms and impairment among girls. These results suggest that when boys experience disordered eating, weight/shape concerns or impairment their likelihood of meeting criteria for an eating disorder is actually greater than that for girls. These data suggest that overvaluation may be a more benign phenomenon in girls, however, when experienced by boys, in particular when it is experienced by boys with mental health vulnerabilities, they are at increased risk of eating disorders.

Unless a patient presents underweight, eating disorders are easily overlooked as a cause of illness in the primary care setting, and given the high prevalence of eating disorders amongst First-Australians, there is a need to screen for eating disorders amongst First-Australians. An opportunity for this may be during Medicare Health Assessments for Aboriginal and Torres Strait Islander People (Medicare Benefits Schedule item 715), which are often conducted in Aboriginal Community Controlled Health Services (ACCHS) by General Practitioners. These are structured health assessments funded by the Australian Government’s universal health scheme, known as Medicare. The cultural and language differences between First-Australians and other-Australians, even amongst those in English-speaking communities suggests the need for a free-to-use, validated and culturally-specific instrument to assist clinicians in screening for eating disorders during these assessments. To our knowledge no such tool has been developed or validated for First-Australians. We suggest such a tool should be developed with First-Australian researchers and communities, similar to the development of the adapted Patient Health Questionnaire (aPHQ-9), a validated and culturally-specific screening tool for depression in First-Australians [29]. Finally, there is currently no evidence describing the uptake of eating disorder treatment for First-Australians. The introduction of Medicare Benefits Schedule items for eating disorders has also highlighted the need for education on eating disorders amongst stakeholders, such as General Practitioners, Nurses, Allied Health personnel and Aboriginal Health Workers, who are most likely to encounter undiagnosed eating disorders. Moreover, we suggest there should be increased awareness of eating disorders amongst school teachers, as they are also likely to encounter students with eating disorders.

This study had several strengths. It was based on data from the EveryBODY study, which was conducted over a broad geographical area including areas with sizable First-Australian populations and differing levels of socioeconomic advantage. The main limitation of this study was that the original data was not gathered with a deliberate intent to investigate eating disorders among First-Australians and not in consultation or collaboration with First-Australian community members or researchers. As such a culturally-informed approach to considering how eating disorders may present in First-Australians was not adopted at the design or data collection phases. On the other hand this specific study utilising the generated data has involved leadership and collaboration from First-Australian researchers (AB, LD), which has allowed for more cultural sensitivity in the interpretation of the existing literature and current findings. As stated above, of major importance in future research in this area would be the development of studies involving First-Australian researchers, that are also grounded within a First-Australian research paradigm as well as methodologies, such as yarning and community-based participation.

## Conclusions

Based on our findings, eating disorders are common amongst First-Australian adolescents, and are associated with poor psychosocial quality of life. These findings are consistent with previous research conducted with First-Australian adults. Given eating disorders occur in the context of poor psychosocial quality of life, which may increase the risk of mental disorders generally, we suggest there is a need to screen for eating disorders in First-Australians. This process may be aided by the development of a validated, culturally specific screening tool for eating disorders in First-Australians. Finally, given the introduction of new MBS items for eating disorders, we suggest there is a need to increase education around the diagnosis and management of eating disorders, especially amongst First-Australians.

## Supplementary information

**Additional file 1: **Supplementary **Table 1.** Operationalisation of DSM-5 Eating Disorder Diagnoses (adapted from Mitchison et al. 2019)

## Data Availability

Data are available from the corresponding author for the purpose of secondary data analyses.

## Abbreviations

χ^2^: Chi Squared test
AAN: Atypical Anorexia Nervosa
ABS: Australian Bureau of Statistics
ACCHS: Aboriginal Community Controlled Health Service
AMS: Aboriginal Medical Service
AN-B: Anorexia Nervosa Broad
aPHQ-9: Adapted Patient Health Questionnaire
ARFID: Avoidant Restrictive Food Intake Disorder
BED: Binge Eating Disorder
BMI: Body Mass Index
BN: Bulimia Nervosa
CI: Confidence Interval
df: Degrees of freedom
DSM-5: Diagnostic and Statistical Manual of Mental Disorders 5th edition
ED: Eating Disorder
EDE-Q: Eating Disorder Examination Questionnaire
FA: First Australian
HOS: Health Omnibus Survey
ICD-11: International Classification of Disease 11th revision
MBS: Medicare Benefits Schedule
MWU: Mann Whitney U test
n: Number of participants
NES: Night Eating Syndrome
OR: Odds Ratio
OSFED: Other Specified Feeding or Eating Disorder
PD: Purging Disorder
PedsQL: Pediatric Quality of Life Scale
SBED: Subthreshold Binge Eating Disorder
SD: Standard Deviation
SEIFA: Socio-Economic Indexes For Areas
UFED: Unspecified Feeding or Eating Disorder
YLD: Years Lived with Disability
