# Supplementary material for: Prevalence, features and health impacts of eating disorders amongst First-Australian Yiramarang (adolescents) and in comparison with other Australian adolescents
Source: J Eat Disord. 2020 Mar 12;8:10. doi: 10.1186/s40337-020-0286-7 (PMC7066723; doi:10.1186/s40337-020-0286-7)
Supplement: Supplementary file 1 — Additional file 1: Supplementary Table 1. Operationalisation of DSM-5 Eating Disorder Diagnoses (adapted from Mitchison et al. 2019) [file 40337_2020_286_MOESM1_ESM.docx]

Supplementary Table 1. Operationalisation of DSM-5 Eating Disorder Diagnoses (adapted from Mitchison et al 2019)

| Diagnosis | Study Criteria |
| --- | --- |
| **Major Eating Disorder** | |
| Anorexia nervosa (AN) | Current BMI percentile < 10; AND persistent extreme weight control behavior (fasting/strict dieting/detox, self-induced vomiting, laxative misuse, driven exercise, or misuse of insulin or other drugs) OR fear of weight gain OR felt fat over the past 4 weeks; AND extreme weight/shape concerns over the past 4 weeks |
| Probable bulimia nervosa (BN) | At least 4 objective binge eating episodes in past 4 weeks; AND persistent extreme weight control behavior in the past 4 weeks (fasting/strict dieting/detox, self-induced vomiting, laxative misuse, driven exercise, or misuse of insulin or other drugs); AND overvaluation of weight and/or shape over the past 4 weeks; AND not meeting criteria for AN |
| Probable binge eating disorder (BED) | At least 4 objective binge eating episodes in past 4 weeks; AND binge eating associated with 3 or more features (rapid eating, eating until uncomfortably full, non-hungry eating, eating alone, feeling disgusted/guilty/depressed after eating); AND marked distress regarding the binge eating; AND absence of persistent extreme weight control behavior over the past 4 weeks (fasting/strict dieting/detox, self-induced vomiting, laxative misuse, driven exercise, or misuse of insulin or other drugs); AND not meeting criteria for AN or BN |
| **Other Specified Feeding and Eating Disorder (OSFED)** | |
| Atypical anorexia nervosa (AAN) | Current BMI percentile > 10; AND lost weight in the past 4 weeks; AND persistent extreme weight control behavior (fasting/strict dieting/detox, self-induced vomiting, laxative misuse, driven exercise, or misuse of insulin or other drugs) OR fear of weight gain OR felt fat over the past 4 weeks; AND extreme weight/shape concerns over the past 4 weeks; AND not meeting criteria for AN or BN or BED |
| Subthreshold bulimia nervosa (SBN) | At least 2 objective binge eating episodes in past 4 weeks; AND at least 2 episodes of extreme weight control behavior in the past 4 weeks (fasting/strict dieting/detox, self-induced vomiting, laxative misuse, driven exercise, or misuse of insulin or other drugs); AND overvaluation of weight and/or shape over the past 4 weeks; AND not meeting criteria for AN or BN or BED |
| Subthreshold binge eating disorder (SBED) | At least 2 objective binge eating episodes in past 4 weeks; AND binge eating associated with 3 or more features (rapid eating, eating until uncomfortably full, non-hungry eating, eating alone, feeling disgusted/guilty/depressed after eating); AND marked distress regarding the binge eating; AND absence of persistent extreme weight control behavior over the past 4 weeks (fasting/strict dieting/detox, self-induced vomiting, laxative misuse, driven exercise, or misuse of insulin or other drugs); AND not meeting criteria for AN or BN or BED |
| Purging disorder (PD) | No binge eating in the past 4 weeks; AND at least 4 episodes of purging in the past 4 weeks (self-induced vomiting, laxative misuse, detox)†; AND not meeting criteria for AN or BN or BED |
| Night eating syndrome (NES) | Night time wakening and eating with awareness at least once/week OR consumption of the majority of daily intake following supper; AND significant psychological distress OR significant functional impairment; AND not meeting criteria for AN or BN or BED |
| **Unspecified Feeding or Eating Disorder (UFED)** | |
| UFED | Persistent binge eating or extreme weight loss behaviour in the past 4 weeks; AND extreme weight/shape concerns over the past 4 weeks; AND significant psychological distress OR significant functional impairment; AND not meeting criteria for AN or BN or BED or OSFED |
| Table adapted from Mitchison D, Mond J, Bussey K, Griffiths S, Trompeter N, Lonergan A, et al. DSM-5 full syndrome, other specified, and unspecified eating disorders in Australian adolescents: prevalence and clinical significance. Psychol Med. 2019:1-10. | |
